# Graphene composites with dental and biomedical applicability

**DOI:** 10.3762/bjnano.9.73

**Published:** 2018-03-05

**Authors:** Sharali Malik, Felicite M Ruddock, Adam H Dowling, Kevin Byrne, Wolfgang Schmitt, Ivan Khalakhan, Yoshihiro Nemoto, Hongxuan Guo, Lok Kumar Shrestha, Katsuhiko Ariga, Jonathan P Hill

**Affiliations:** 1 Institute of Nanotechnology, Karlsruhe Institute of Technology (KIT), D-76131 Karlsruhe, Germany; 2 Department of Civil Engineering, Liverpool John Moores University, Byrom Street, Liverpool, L3 3AF, United Kingdom; 3 Materials Science Unit, Division of Oral Biosciences, Dublin Dental University Hospital, Trinity College Dublin, Lincoln Place, Dublin 2, Ireland; 4 School of Chemistry and CRANN Institute, University of Dublin, Trinity College, Dublin 2, Ireland; 5 Charles University, Faculty of Mathematics and Physics, Department of Surface and Plasma Science, V Holešovičkách 2, 18000 Prague 8, Czech Republic; 6 International Center for Materials Nanoarchitectonics (WPI-MANA), National Institute for Materials Science (NIMS), Namiki 1-1, Tsukuba, Japan; 7 Department of Advanced Materials Science, Graduate School of Frontier Sciences, The University of Tokyo, 5-1-5 Kashiwanoha, Kashiwa, Chiba 277-8561, Japan

**Keywords:** biocompatibility, bioglass, graphene, mechanical properties, nanocomposite

## Abstract

Pure graphene in the form of few-layer graphene (FLG) – 1 to 6 layers – is biocompatible and non-cytotoxic. This makes FLG an ideal material to incorporate into dental polymers to increase their strength and durability. It is well known that graphene has high mechanical strength and has been shown to enhance the mechanical, physical and chemical properties of biomaterials. However, for commercial applicability, methods to produce larger than lab-scale quantities of graphene are required. Here, we present a simple method to make large quantities of FLG starting with commercially available multi-layer graphene (MLG). This FLG material was then used to fabricate graphene dental-polymer composites. The resultant graphene-modified composites show that low concentrations of graphene (ca. 0.2 wt %) lead to enhanced performance improvement in physio-mechanical properties – the mean compressive strength increased by 27% and the mean compressive modulus increased by 22%. Herein we report a new, cheap and simple method to make large quantities of few-layer graphene which was then incorporated into a common dental polymer to fabricate graphene-composites which shows very promising mechanical properties.

## Introduction

Now that much of the world’s population are living beyond their “threescore years and ten” [1], that is to say, on average, into their 80s [2], there has been an increase in the need for minimal intervention dentistry [3]. This practice of a complete management solution for tooth decay has benefited from the extensive use of dental polymers. However, current dental polymers have a relatively short operational lifetime resulting from their lack of sufficient strength and durability. Therefore, the aim was to assess the use of graphene with a common dental polymer to form a composite material with improved mechanical properties.

One of the main problems facing dental-polymers is that of location. They are situated within the mouth which is an extremely demanding setting – exposure to moisture, high temperatures, and abrasion from toothbrushes plus a variety of foodstuffs all have to be dealt with. These conditions can lead to problems of mechanical failures cancelling out initial clinical success and over time requiring further work for restoration with the associated inconvenience and extra cost. Then there is the issue of biocompatibility to consider.

Biocompatibility is a prerequisite for all dental materials. They must be compatible with oral fluids, must not release toxic products into the oral location and must have sufficient strength and durability to be fit for purpose [4]. Most other studies of graphene-dental polymer materials have used graphene oxide (GO) [5] which may be cytotoxic [6–7]. Therefore, in these tests glass-ionomers (GIs) prepared with poly(acrylic acid), a common dental polymer [8], were used with the addition of few-layer graphene (FLG). Graphene has the advantages of having a high fracture and mechanical strength, a large surface area, flexibility and is also biocompatible and thought to be non-cytotoxic [9–13], but as toxicity depends on many factors such as size, shape, concentration and dose further studies with regard to specific applications are needed. Therefore, the aim of these experiments was to assess the use of graphene with a glass-ionomer (GI) prepared with poly(acrylic acid) to form a biocompatible composite material with improved mechanical properties.

## Results and Discussion

### Few-layer graphene

**Figure 1:**
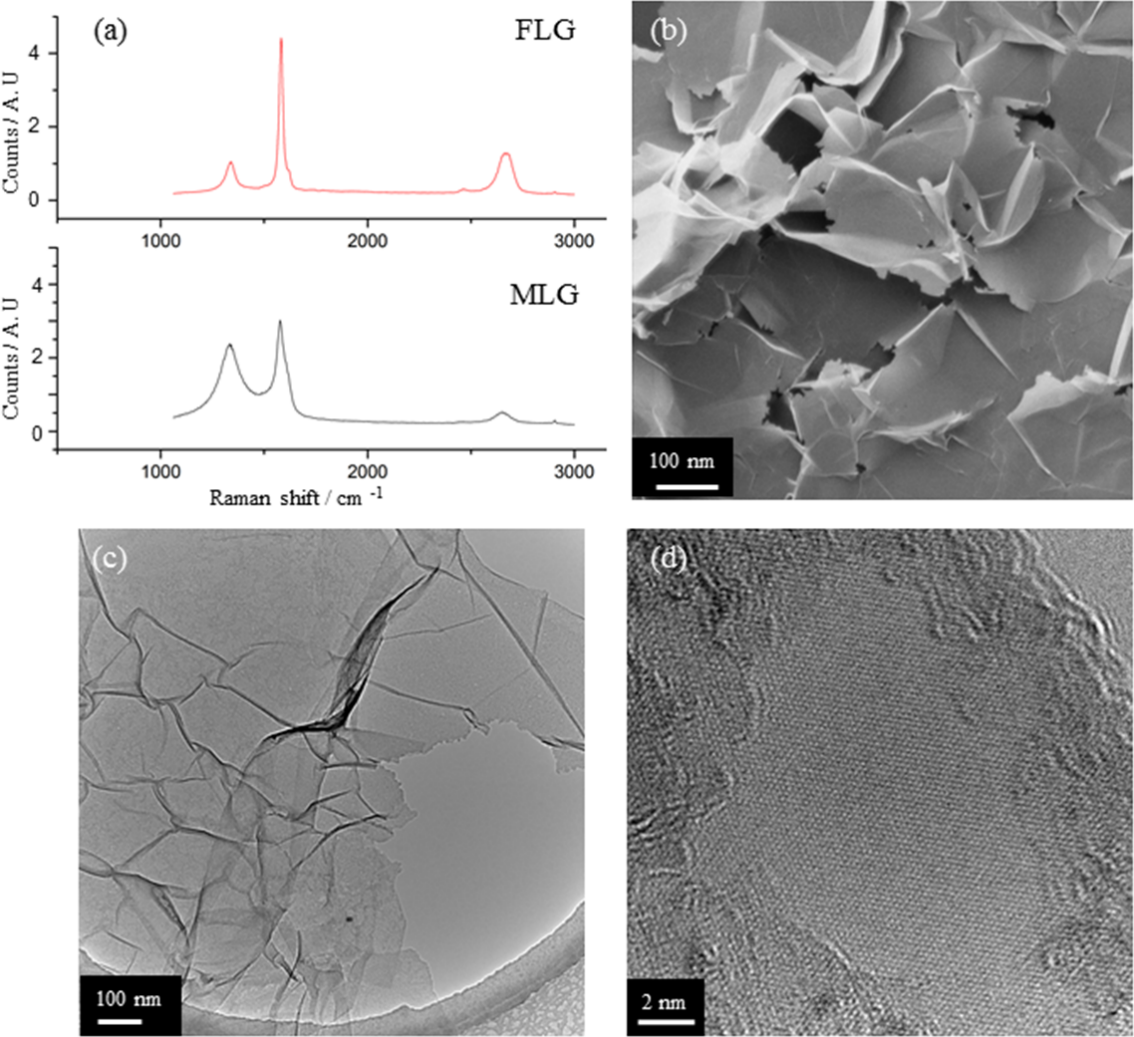
a) Raman spectra of MLG (ca. 10 layers, lower) and FLG (1–6 layers, upper) – both at 514 nm. b) Helium ion microscope (HeIM) overview of FLG, c) TEM overview of FLG and d) HRTEM detail of FLG showing a single layer.

For the graphene material it was decided to use commercially available multi-layer graphene (MLG) from Graphit Kropfmühl GmbH (EXGR98350 - batch 08.10.2012). The shape and position of the Raman 2D band (≈2700 cm^−1^) provides a useful analysis for assessing the quality and number of layers in graphene materials [14–15]. As the FLG material is composed of “flakes” the edges of the flakes give rise to a D band. The shape and position of the 2D band in the MLG material is indicative of multi-layer graphene rather than graphite and the 2D band in the FLG material is indicative of few-layer graphene [14]. This allows us to see the conversion of commercial MLG material (Figure 1a, lower) to FLG (Figure 1a, upper, 1b–d).

**Figure 2:**
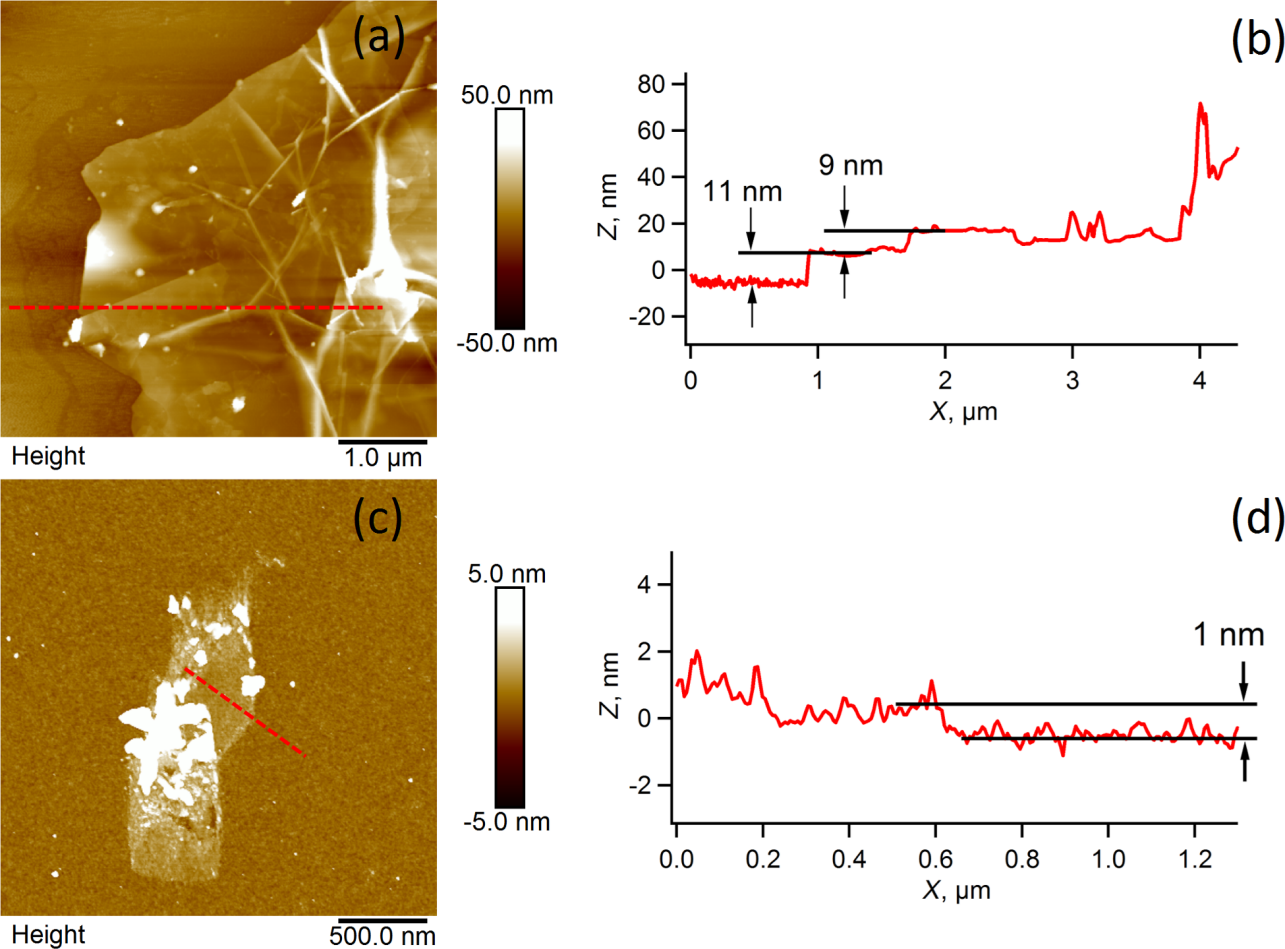
a) and b) AFM detail and profile of a multi-layer graphene (MLG) flake, ca. 10 graphene layers, c) and d) AFM detail and profile of a few-layer graphene (FLG) flake, ca. 1–6 graphene layers.

Figure 2 shows AFM (detail and profile) of the graphene material (MLG) before and (FLG) after heat-treatment in air at 500 °C for 2 h (ca. 10% volume loss of starting mass during heat-treatment). This resulted in the FLG material used in these experiments.

The XPS analysis (Table 1) shows that the MLG and FLG materials have similar oxygen content as the O 1s/C 1s ratios are very similar. The binding energies (*E*_b_) ≈284.6 eV corresponds to C–H, C–C, (CH_2_)*_n_* and C=C bonds that are characteristic of graphite/graphene, ≈286 eV corresponds to C–O–C, ≈288.5 eV corresponds to O–C=O, ≈531.5–532 eV corresponds to C–O and ≈533 eV corresponds to C=O [16]. Therefore, it is reasonable to assume that the loss of carbon and oxygen from the MLG material compared to the FLG material can be attributed to the formation of CO and CO_2_ during the heat-treatment. This is in accord with the Raman data which shows a clear “finger-print” for graphene rather than graphene oxide [14–15].

**Table 1:** Chemical composition of MLG and FLG from XPS analysis.

	Component	*E*_b_ (eV)	FWHM (eV)	Area (eV)	Fraction (%)	O 1s/C 1s

MLG	C 1s	284.01	1.83	8649.91	60.00	0.130
	C 1s	285.39	3.09	3151.94	21.87	
	C 1s	289.44	3.37	736.77	5.12	
	O 1s	532.39	2.36	4202.32	10.61	
	O 1s	530.44	2.07	188.13	0.75	
	N 1s	399.36	2.07	188.13	0.75	
FLG	C 1s	283.67	1.58	8588.15	56.18	0.170
	C 1s	284.83	1.94	3097.75	20.27	
	C 1s	285.62	3.01	1254.09	8.21	
	O 1s	532.43	1.58	1384.56	3.30	
	O 1s	531.86	2.86	4686.88	11.15	
	N 1s	399.35	2.48	238.66	0.89	

### FLG-dental polymers

**Figure 3:**
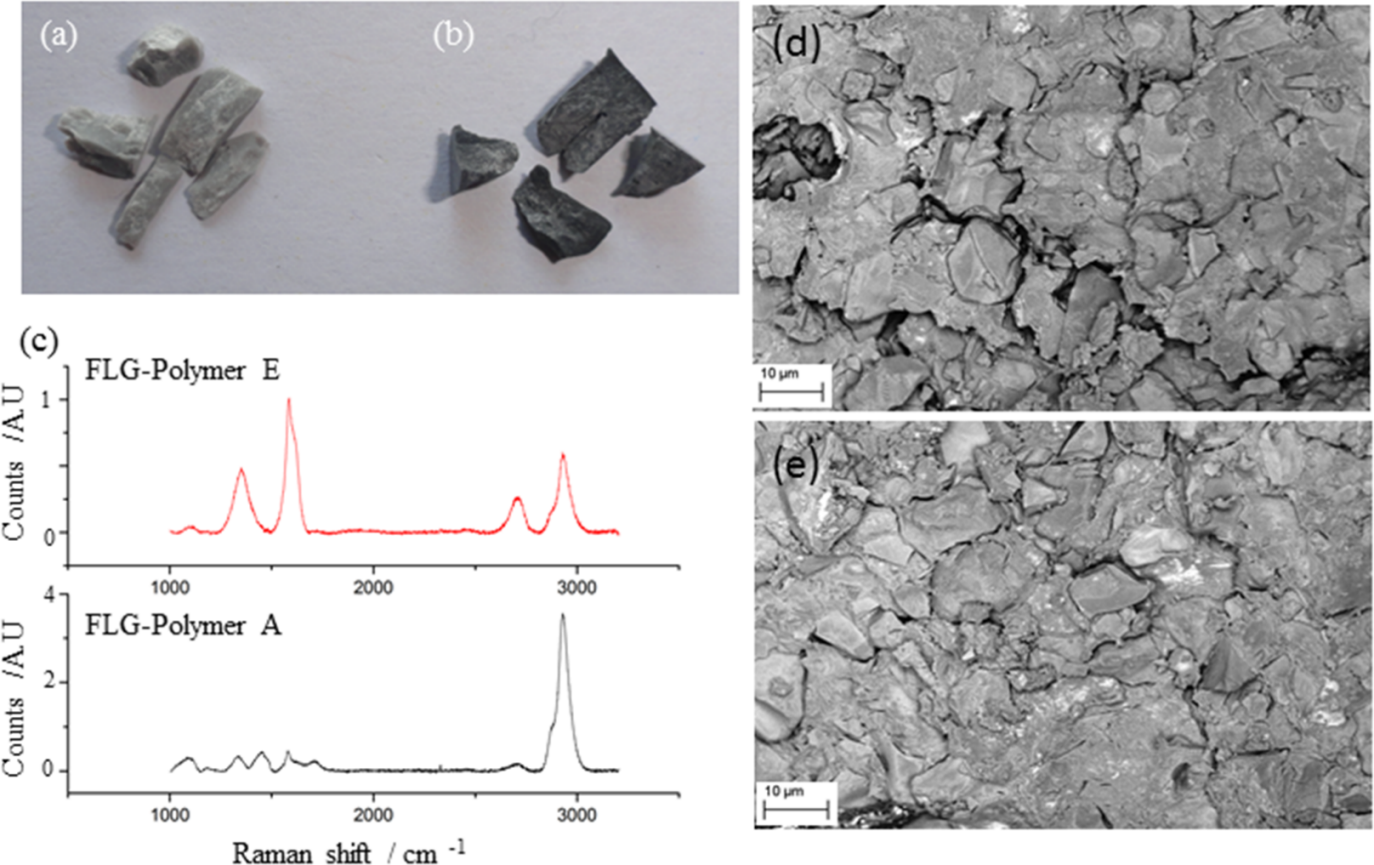
a) GI composite after strength testing made from FLG-polymer A, b) GI composite after strength testing made from FLG-polymer E, c) Raman spectra of GI composite made from FLG-polymer E and GI composite made from FLG-polymer A – both at 514 nm, d) SEM overview of fracture surface of GI composite made from FLG-polymer A, e) SEM overview of fracture surface of GI composite made from FLG-polymer E.

Six types of FLG-dental polymers were made up; one control plus five with different loadings of graphene. Figure 3 shows FLG-polymer A (lowest concentration of FLG) and FLG-polymer E (highest concentration of FLG used), hence E appears much darker than A (Figure 3a,b).

The Raman spectra of both FLG-dental polymers show a 2D band (≈2700 cm^−1^) which is indicative of FLG [14–15] although in the higher graphene loaded polymer this band is more pronounced. The fracture sections of both polymers were conducting enough to need no coating to be examined by SEM. This is indicative of a good percolation network of the FLG material in the dental polymer (Figure 3d and 3e). The SEMs were obtained using an energy selective backscatter (EsB) detector which gives clear compositional contrast. In these micrographs the white patches correspond to graphene in the fracture surface of the polymer matrix.

The mean dynamic viscosity, compressive fracture strength and compressive modulus and associated standard deviations for the control group and the groups prepared with poly(acrylic acid) solutions containing graphene are shown in Table 2.

**Table 2:** The mean dynamic viscosity, compressive fracture strength and compressive modulus ± standard deviation for the control group and the groups prepared with poly(acrylic acid) solutions containing graphene.

Group	Dynamic viscosity (mPa·s)	Compressive fracture strength (MPa)	Compressive modulus (GPa)

Control	610 ± 0	93.3 ± 4.6	2.91 ± 0.12
A – 0.5 mg	617 ± 6	118.2 ± 8.3	3.56 ± 0.32
B – 1.0 mg	623 ± 6	111.3 ± 5.2	3.32 ± 0.11
C – 2.0 mg	653 ± 6	116.5 ± 7.8	3.49 ± 0.10
D – 5.0 mg	680 ± 10	111.0 ± 5.8	3.16 ± 0.15
E – 10.0 mg	713 ± 6	105.3 ± 7.1	3.18 ± 0.09

There was a progressive significant increase in the dynamic viscosity of the poly(acrylic acid) solutions as the concentration of graphene added to the poly(acrylic acid) solutions was increased. This increase in viscosity with increasing nano-carbon concentration is consistent with that found by other researchers [17–18]. Further increases in the amount of graphene added to the poly(acrylic acid) solutions – 2.0 mg, 5.0 mg and 10.0 mg all resulted in significant increases in dynamic viscosity compared with the control group as illustrated in Table 2.

**Figure 4:**
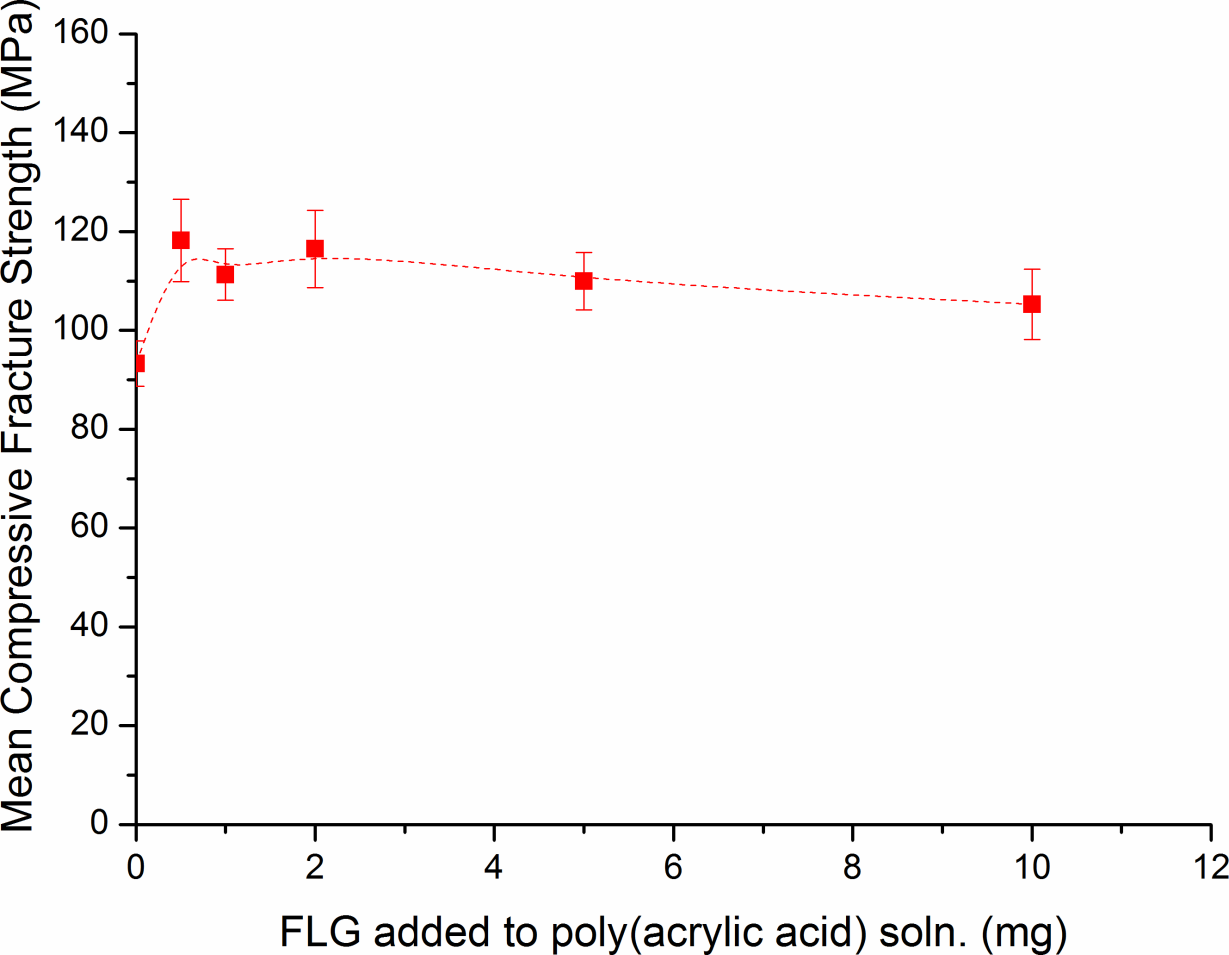
Change in mean compressive fracture strength with increasing graphene concentration.

There was no significant trend in the compressive fracture strength data with increasing concentration of graphene added to the poly(acrylic acid) solutions as shown in Figure 4. The group prepared using a poly(acrylic acid) solution containing 0.5 mg of graphene produced the highest mean compressive fracture strength (118.2 ± 8.3 MPa) which was a 27% increase compared with the control group (93.3 ± 4.6 MPa).

**Figure 5:**
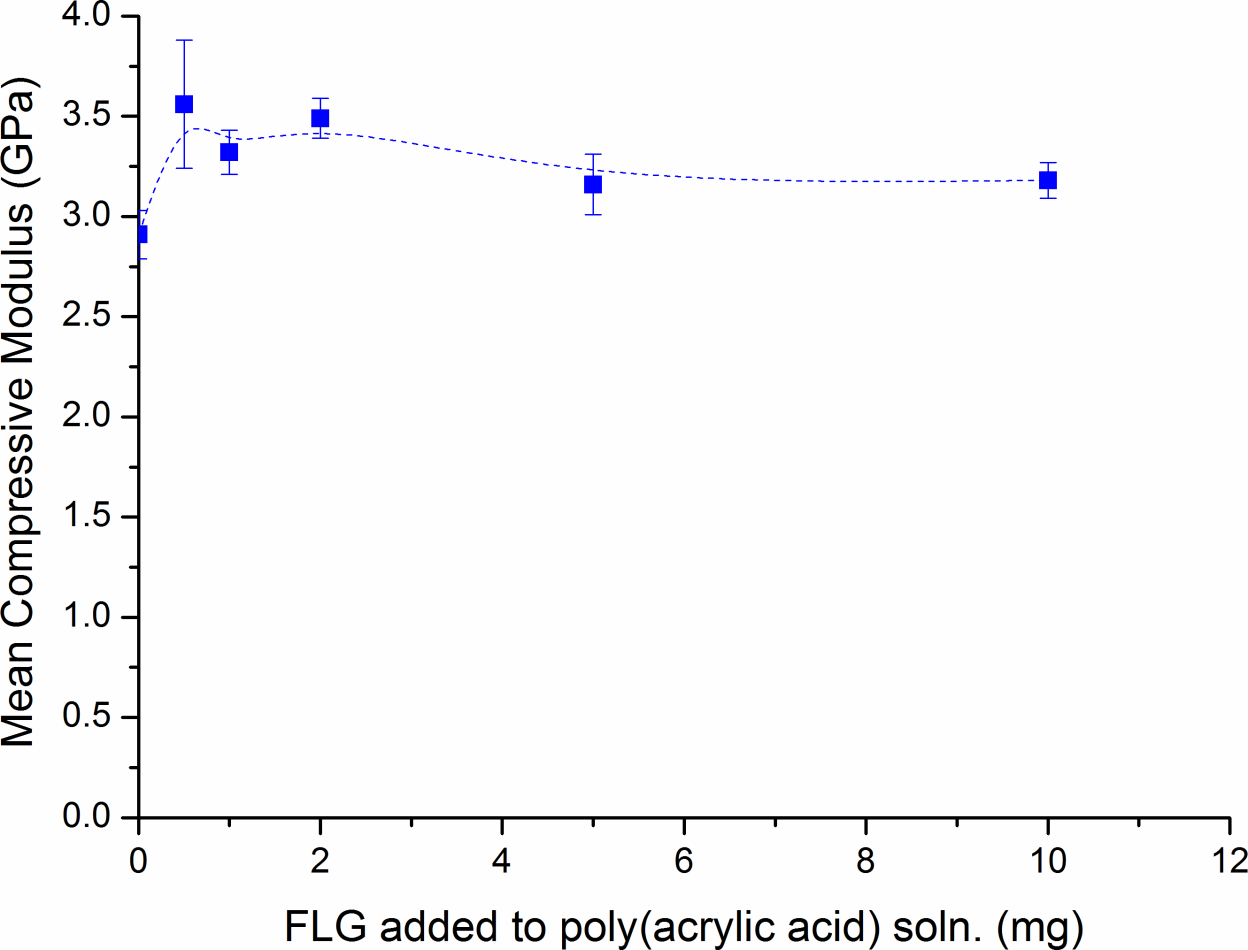
Change in mean compressive modulus with increasing graphene concentration.

For the compressive modulus data, there was no significant trend as the concentration of graphene added to the poly(acrylic acid) solutions was increased as shown in Figure 5. Significant increases in the compressive modulus data were reported for all groups prepared with poly(acrylic acid) solutions containing graphene compared with the control group as shown in Table 2. Similarly to the results from the compressive fracture strength data, the group which produced the highest mean compressive modulus (3.56 ± 0.32 GPa) was the group containing 0.5 mg of graphene, which showed a 22% increase compared with the control group (2.91 ± 0.12 GPa).

From the results of the mechanical testing it is clear that a small addition of FLG gives a large increase in the FLG-dental polymer fracture strength and compressive modulus. The latter is significant as it shows the capacity of the FLG-dental polymer to withstand loads tending to reduce in size – e.g., biting and chewing. The decrease in these enhanced properties with increasing FLG loading is probably due to aggregation of the FLG in the polymer matrix. Further optimisation tests are ongoing.

## Conclusion

In summary, we have described herein a new, simple and cheap method to make large quantities of FLG starting with commercially available multi-layer graphene (MLG) and also the incorporation of this graphene into dental polymer composites. We have demonstrated that the fabricated graphene-dental polymer composites have significantly enhanced mechanical properties as compared with the plain dental-polymer material (control group). The mean compressive strength of the graphene-dental polymer showed a 27% increase and the mean compressive modulus showed a 22% increase compared with the control group – this is a significant increase. A recent review [8] concluded that despite the developments in GI powder and poly(acrylic acid) constituents they still had inferior mechanical properties compared with dental amalgam and resin based composites. They go on to say that major improvements have yet to be made so that GIs can see real clinical usage. Therefore, these studies could well be the “major improvements” sought for as they show that GIs with a low concentration of graphene lead to major performance improvement in physio-mechanical properties. This represents a major advance in GI materials reinforcement strategy and will breathe a new lease of life into this research area. These in vitro studies are continuing and cell-line studies are also planned.

## Experimental

### Materials

The multi-layer graphene (MLG) material used in this research was commercially available MLG - EXGR98350 (batch 08.10.2012) supplied by Graphit Kropfmühl GmbH (Hauzenberg, Germany). The MLG material was heated in air at 500 °C for 2 h to give the FLG material.

### Graphene-polymer composite preparation

Poly(acrylic acid) powder, 1.0 g, with an average molecular weight of 40000 was mixed with 2.5 mL of distilled water to give a concentration of 40%. Then five concentrations of FLG and these poly(acrylic acid) solutions were made up containing 0.5, 1.0, 2.0, 5.0 or 10.0 mg of FLG (Table 2, group A–E). A control poly(acrylic acid) solution was also prepared without graphene by dissolving 1.0 g of the poly(acrylic acid) powder in 2.5 mL of distilled water (Table 2, group control). All the solutions were sonicated for 15 min and then stirred for 24 h.

All the poly(acrylic acid) solutions (A–E and control) were hand-mixed with a commercial glass-ionomer (GI) restorative powder (Ionofil Molar; Voco GmbH, Cuxhaven, Germany) using a powder to liquid mixing ratio of 4:1 (g/g) as recommended by Voco GmbH. In each case 0.188 g of the poly(acrylic acid) solution was pipetted onto one end of a glass slab while 0.75 g of the Ionofil Molar powder was placed onto the opposite end. The GI powder was divided into two halves, the first half was hand-mixed with all the poly(acrylic acid) solution for 20 s using a stainless steel spatula, and then the remaining GI powder was added and mixed for a further 20 s.

### Dynamic viscosity measurements

The viscosity of all the poly(acrylic acid) solutions was measured with a digital viscometer (Brookfield DV-E Viscometer; Brookfield Engineering Laboratories Inc., Middleboro, MA, USA). The poly(acrylic acid) solution was pipetted into the inner chamber of a small sample adaptor attached to the viscometer and a spindle was inserted slowly into the chamber to avoid entrapping air bubbles in the poly(acrylic acid) solution. The spindle was rotated in the poly(acrylic acid) solution at 100 rpm until a constant viscosity reading was obtained and the dynamic viscosity (mPa·s) was recorded. In total, three viscosity measurements were taken for each of the poly(acrylic acid) solutions and the mean dynamic viscosity calculated.

### Compressive fracture strength tests

The compressive fracture strength was determined by preparing cylindrical specimens of 6.0 ± 0.1 mm height and 4.0 ± 0.1 mm diameter in accordance with ISO 9917-1 [19] using a Teflon split-mould [20]. The split-mould was placed on a Teflon base covered with an acetate strip and aligned using nylon wedges and a locating pin. The hand-mixed GI restorative plastic mass was applied to one side of the split-mould immediately after mixing using the stainless steel spatula and allowed to flow into the mould to minimise air bubble incorporation in the set cylindrical specimens. A second acetate strip was placed on top of the filled mould and the whole mould assembly was isolated from the surrounding atmosphere using a glass-slab and a G-clamp before transfer to a water-bath maintained at 37 ± 1 °C. After 1 h in the water bath, the specimens were removed from the mould, inspected and specimens containing visual defects were discarded. The flat ends of the specimens were hand-lapped on P600 silicon carbide paper (Beuhler, Lake Bluff, Illinois, USA) under water lubrication to ensure parallel specimen ends for uniform contact with the platens of the testing apparatus [21]. The specimens were stored in glass containers filled with 50 mL of distilled water in an incubator at 37 ± 1 °C for a further 23 h prior to testing. Ten nominally identical cylindrical GI restorative specimens were manufactured for each group investigated.

The mean diameter of each specimen was determined from three measurements taken using a digital micrometer accurate to 10 μm (Mitutoyo, Kawasaki, Japan). The compressive fracture strength of each specimen was made by applying a compressive load to the long axis of the specimen at a cross-head speed of 1 mm/min using a tensile testing apparatus (Instron Model 5565, High Wycombe, England). In order to mimic the oral environment, wet filter paper was placed on the flat ends of the specimen prior to testing [19]. The compressive fracture strength P (MPa) was calculated using Equation 1 [19],

[1]
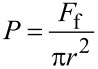


where *F*_f_ was the load at fracture (N) and *r* the mean radius of the specimen (mm). The change in stress Δσ (MPa) and strain Δε generated in each specimen during compression testing was quantified using Equation 2 and Equation 3, respectively.

[2]
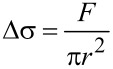


[3]
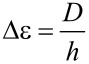


where *F* was the load (N), *r* the original mean radius of the specimen (mm), *D* the deflection undertaken by the specimen during testing (mm) and *h* the original height of the specimen (mm). Stress/strain plots were derived for each individual specimen and the compressive modulus (the ratio of stress to strain below the fracture limit) was determined by calculating the slope of the initial straight portion of the stress/strain plot prior to fracture [20].

### Statistics

All data in Table 1 are presented as means ± SD and were derived from ten independent samples at each FLG concentration. The one-way ANOVA (*p* < 0.0001) and Tukey’s post-hoc tests of the compressive fracture strength data identified significant increases for all the groups containing FLG compared with the control group (*p* = 0.003). For the compressive modulus data, the one-way ANOVA (*p* = 0.0001) and Tukey’s post hoc tests also identified significant increases for all the groups containing FLG compared with the control group (*p* = 0.003).

### Characterization

The MLG and FLG material was characterized by Raman spectroscopy (Renishaw at 514 nm) and the AFM measurements were performed on a MultiMode V AFM (Veeco) in tapping mode under ambient conditions. RTESP silicon probes (Veeco) were used with a nominal tip radius of 10 nm and nominal spring constant of 40 N/m. Image processing was carried out using the Nanoscope software. The X-ray photoelectron spectroscopy (XPS) measurements were performed on a Theta Probe spectrometer (Thermo Electron Co., Germany) using monochromatic Al Kα radiation (photon energy of 15 keV with maximum energy resolution of 0.47 eV). High resolution spectra for the core level C 1s and O 1s were recorded in 0.05 eV steps. An electron flood gun was used during the measurements to prevent sample charging. The FLG material was also characterized by TEM, HRTEM (Jeol ARM at 80 kV) and helium ion microscopy (HeIM, Zeiss Orion at 30 kV). In addition, FLG-polymer A and E were characterized by Raman Spectroscopy (JY T6400 at 514 nm) and SEM (Zeiss Ultra-Plus at 3 kV, EsB grid at 503 V).
